# Developing Public‐Friendly Visualisations to Improve PPIE Glossaries for Statistical Methodology Research

**DOI:** 10.1111/hex.70690

**Published:** 2026-05-14

**Authors:** Clareece R. Nevill, Hannah Cooper, Barbara Czyznikowska, Louise Fairgrieve, Justin Greenwood, Gurpreet Grewal‐Santini, Janion Nevill, Lucy Teece, Molly Wells, Suzanne C. Freeman, Sarah Booth

**Affiliations:** ^1^ Biostatistics Research Group, Division of Public Health and Epidemiology, School of Medical Sciences University of Leicester Leicester UK; ^2^ Centre for Ethnic Health Research University of Leicester Leicester UK; ^3^ Institute for Precision Health, School of Medical Sciences University of Leicester Leicester UK; ^4^ Public Contributor, PPI‐SMART (Patient and Public Involvement for Statistical Methodology and Research Techniques) University of Leicester Leicester UK; ^5^ AstraZeneca Macclesfield UK; ^6^ Division of Informatics, Imaging and Data Science, Faculty of Biology, Medicine and Health University of Manchester Manchester UK

**Keywords:** Patient and Public Involvement and Engagement (PPIE), statistical methodology, visualisations

## Abstract

**Background:**

Plain‐language definitions help patients and the public engage in research, but some technical terms are easier to understand with visual aids. Furthermore, many people find visual aids more intuitive when learning new concepts. The PPI‐SMART group at the University of Leicester recently created a plain‐language glossary for statistical methodology research terms. This project aimed to develop visualisations for selected glossary terms to support Patient and Public Involvement and Engagement (PPIE) in statistical methodology research, where such resources are scarce.

**Methods:**

We selected 10 glossary definitions to develop visualisations. The working group sketched initial ideas, which a graphic designer developed into drafts. After refinement, the visualisations underwent two cycles of feedback from public contributors. Suggestions were incorporated through the cycles until a final set of visualisations was created.

**Results:**

Visualisations were created for the terms: Bayesian, calibration, causal inference, censoring, deviance, discrimination, regression, Markov Chain Monte Carlo (MCMC), prognostic model, and simulation study. Feedback highlighted issues such as alternative interpretations of language, layout simplicity, accessibility needs and symbol interpretation. These insights shaped the final designs.

**Conclusions:**

Resources to support PPIE in statistical methodology research are limited but needed. We developed 10 visualisations to help PPIE members understand complex terminology. These are freely available on the NIHR Leicester Biomedical Research Centre website: https://leicesterbrc.nihr.ac.uk/ppismart/ppismart-definitions/.

**Patient or Public Contribution:**

Two groups of public contributors reviewed the visualisations. Their feedback, received during online meetings and via email, ensured the visuals met the aim of making statistical terms easier to understand.

## 1 Introduction

The process of developing, refining and improving statistical tools that are used in health‐related studies and research—known as statistical methodology research—is an essential component for improving health outcomes. Advances in statistical methodology have long played a critical role in improving health research, from enhancing disease surveillance and causal inference [1] to strengthening the design and analysis of clinical trials [2]. Furthermore, output from statistical methodology research is often used within health technology assessments and clinical guidelines, ultimately shaping healthcare policy and improving how healthcare is provided. Patient and Public Involvement and Engagement (PPIE) is already known to benefit health research, from improving study design and recruitment, to helping interpret and communicate results [3]. Whilst some disagree on the importance of PPIE for statistical methodology research, as with health research, the incorporation of PPIE ensures that the output is relevant and applicable to as many people as possible [4]. An example of the benefit of PPIE in statistical methodology research can be seen when a statistical model for understanding the natural history of Duchenne Muscular Dystrophy was being developed—through PPIE activities, an additional progression stage was identified, thus creating a more representative and accurate model [5]. Furthermore, as organisations in the United Kingdom such as the National Institute for Health and Care Research (NIHR) and the Medical Research Council (MRC) are funded from public money, there is a moral argument for PPIE to be a mandatory element for research that is funded through these streams [6].

Many researchers working in the field of statistical methodology research would feel more confident to conduct PPIE in their work if there were more resources available specifically designed for statistical methodology research [4]. To this end, the PPI‐SMART (PPI for Statistical Methodology and Research Techniques) group, based at the University of Leicester, has been developing freely available resources to improve the incorporation of PPIE in statistical methodology research. Projects have included developing an introductory animation [7], case studies, workshops [8], and a plain‐language glossary [9] (https://leicesterbrc.nihr.ac.uk/ppismart). When developing the glossary of terms commonly used in statistical methodology research, it was found that some terms would benefit from having a visualisation accompanying the definition.

In various areas of life, visualisations are found to be beneficial—we all know the phrase ‘a picture speaks a thousand words’. They are often preferred over large blocks of text since visual information can be processed quicker, leading to reduced cognitive load. In addition to being more engaging, visualisations can help audiences of varying backgrounds comprehend the topic [10].

### 1.1 Aims of Project and PPIE

The aim of this project is to develop and introduce public‐friendly visualisations to complement some of the previously developed statistical methodology research definitions. The aim of the PPIE within this project was to gain feedback on the proposed visualisations to ensure that they would be suitable, understandable and accessible to a broad range of PPIE members taking part in future statistical methodology research projects.

## 2 Materials and Methods

### 2.1 Setting

This project was built on our previous work of developing a plain‐language glossary for public contributors taking part in PPIE activities related to statistical methodology research [9]. The glossary contained 64 commonly used statistical terms covering a range of concepts, including statistical models, Bayesian analysis, meta‐analysis and prognostic modelling. During development of the glossary, public contributors highlighted that they would find having a visualisation alongside the definition useful in helping their understanding of the terms. One of the terms, ‘Bayesian’ (which refers to a specific way of doing statistics, by learning and making our predictions better using new information), was highlighted as a term that would lend itself well to having a complementary visualisation. Unfortunately, during glossary development, creating accompanying visualisations was not possible due to funding constraints; however, additional funding was later obtained to develop a set of visualisations for 10 of the definitions in our glossary (including ‘Bayesian’, with the remaining nine to be decided).

### 2.2 Research Team

This was a collaborative project which ran from April 2024 to September 2025. The team included a working group of researchers from the PPI‐SMART group (University of Leicester) and the Centre for Ethnic Health Research, a graphic designer (University of Leicester) and two PPIE Groups (recruited through the PPI‐SMART group and the Centre for Ethnic Health Research, respectively). Both PPIE groups comprised five public contributors with a mixture of protected characteristics (e.g., age, sex and race) and included individuals for whom English was not their first language. Both groups had been involved in the development of the glossary and were therefore already familiar with the terms and definitions.

### 2.3 Development Process

**Figure 1:**
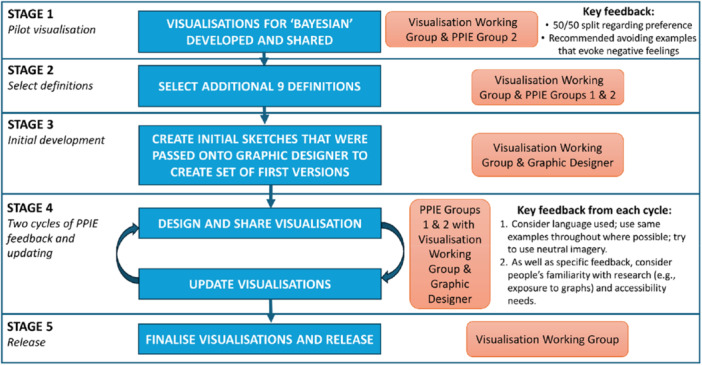
Visual diagram explaining the process for developing the visualisations.

The process of developing the visualisations for the glossary consisted of five stages and is summarised in Figure 1. The GRIPP2 (Guidance for Reporting Involvement of Patients and the Public) checklist [11] was followed and completed (see Additional file 1).

#### 2.3.1 Stage 1: Piloting a Visualisation

As an initial step, two visualisations for the term ‘Bayesian’ were developed by the graphic designer. ‘Bayesian’ was identified during the glossary development as a starting point by the working group, as they felt it was a term that would lend itself to a visual definition much better than a written one. The two visualisations were discussed with PPIE Group 2 to determine which visualisation they preferred and if they had any general feedback and recommendations for developing the visualisations.

#### 2.3.2 Stage 2: Selection of an Additional Nine Definitions

The term ‘Bayesian’ was already selected as being one of the 10 chosen definitions before funding was secured. The decision of which remaining definitions to develop visualisations for was based on:

1. Feedback from PPIE Groups 1 and 2 about which terms they felt would benefit most from a visualisation.
2. Feedback from the working group about which terms they felt could be explained well visually.
3. Which terms had been highlighted as being complex or difficult to understand during the PPIE meetings for the development of the glossary.

#### 2.3.3 Stage 3: Initial Development of Sketches

The working group developed pen‐and‐paper sketches for each of the remaining nine terms (i.e., not ‘Bayesian’), which were developed into digital visualisations by the graphic designer. Along with the chosen visualisation from the pilot stage for ‘Bayesian’, this gave an initial set of 10 visualisations. The working group gave feedback on these visualisations, which led to an updated set of visualisations (which included multiple options for some terms).

#### 2.3.4 Stage 4: Cycles of PPIE Feedback

Any visualisations that were deemed as complex by the working group, or had multiple options, were discussed online with four members of PPIE Group 1. The primary aim of this round of PPIE feedback was to gain feedback to help decide which visualisations to take forward.

Using the feedback from this meeting, we updated the visualisations and held an online meeting with PPIE Group 2 to discuss all 10 visualisations. To reduce workload but ensure every visualisation was seen by two people ahead of the meeting, each public contributor was emailed four different visualisations from the set of ten. After implementing the feedback from this meeting, the visualisations were finalised.

Whilst the primary aim of the PPIE was to gain feedback on the visualisations, for some terms, this led to discussions and further feedback on the wording of the definitions. These definitions were subsequently updated.

#### 2.3.5 Stage 5: Release

The visualisations have been made freely available on the NIHR Leicester BRC website for researchers to download and use (https://leicesterbrc.nihr.ac.uk/ppismart/ppismart-definitions/).

## 3 Results

### 3.1 Stage 1: Piloting a Visualisation

**Figure 2:**
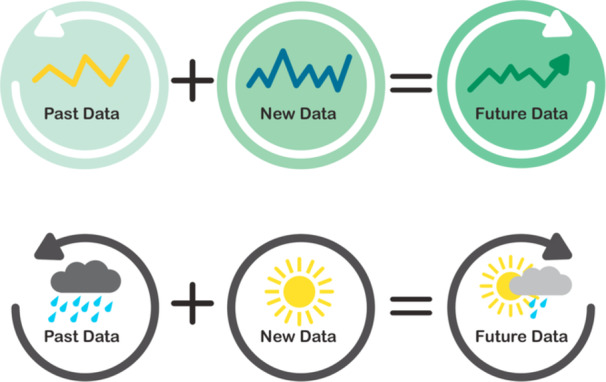
Pilot visualisations for ‘Bayesian’.

Four members from PPIE Group 2 discussed the two visualisations for the term ‘Bayesian’ (Figure 2). One used a weather‐themed example which showed how past (heavy rain) and present (sunny) weather conditions could be combined to predict the future weather being partially sunny and cloudy. The second visualisation used graphs of old and new data, and the future data was a combination of the two. There was a 50/50 split as to which visualisation was preferred, with some noting that the rain invoked a negative feeling in the weather‐themed visualisation and that in the graph example the trends in the data were not distinct enough. Recommendations were made to avoid using examples which invoked negative feelings and to ensure that each image includes a title so it is clear which term is being visualised. The weather theme was chosen for the next iteration of the visualisation, along with changes as per the group's recommendations.

### 3.2 Stage 2: Selection of 10 Definitions

Each public contributor from PPIE Group 1 was asked to email us with the five terms that they felt would benefit most from a visualisation. Perhaps due to there being 64 terms in the glossary, there was very little overlap between the responses, except for ‘causal inference’ and ‘regression’, which were both selected by two public contributors. Other terms which were suggested included ‘deviance’, ‘Markov Chain Monte Carlo (MCMC)’ and ‘simulation study’.

Feedback from PPIE Group 2 was that it would be useful to prioritise definitions which have an alternative meaning in everyday language, such as ‘discrimination’ (of a prognostic model), ‘censoring’ (survival analysis) and ‘deviance’ (model fit).

Amongst the working group, it was felt that ‘regression’ and ‘deviance’ were quite visual concepts that could be explained well through a graph. Furthermore, the glossary definition of ‘Markov Chain Monte Carlo (MCMC)’ used imagery of taking random steps within a maze which could be incorporated into a visualisation.

Since it was recommended by PPIE Group 2 to include a visualisation for ‘discrimination’, it was felt that it would be useful to also develop a visualisation for ‘prognostic model’ as this is central to understanding the concept of discrimination. Calibration was also selected since this is another important concept in prognostic modelling.

Finally, it was felt that ‘simulation study’ was a term that was difficult to explain in the glossary and therefore a visualisation may help to further explain the concept.

In summary, the 10 selected terms were: Bayesian, calibration, causal inference, censoring, deviance, discrimination, regression, Markov Chain Monte Carlo (MCMC), prognostic model, and simulation study.

### 3.3 Stage 3: Initial Development of Sketches

**Figure 3:**
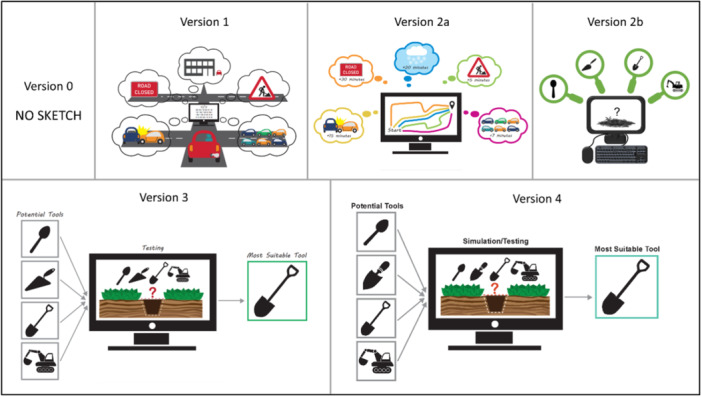
Progression of visualising the term ‘simulation study’.

**Figure 4:**
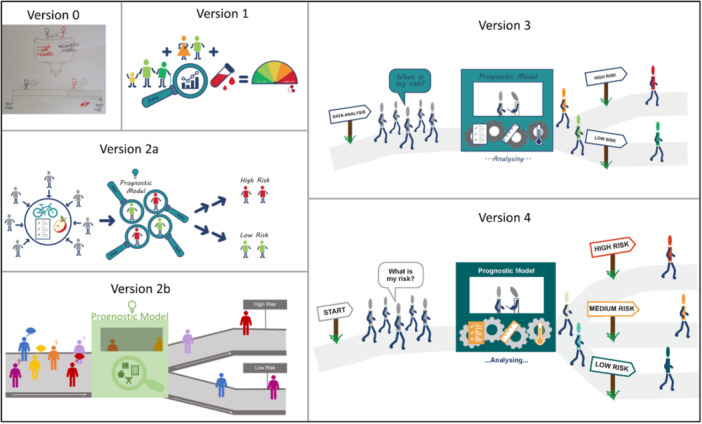
Progression of visualising the term ‘prognostic model’.

**Figure 5:**
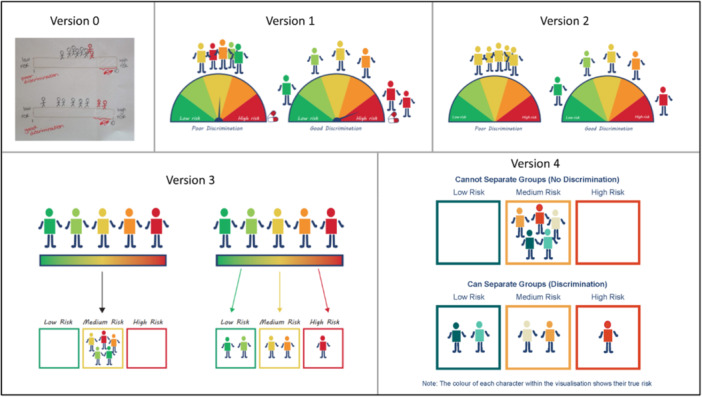
Progression of visualising the term ‘discrimination’.

**Figure 6:**
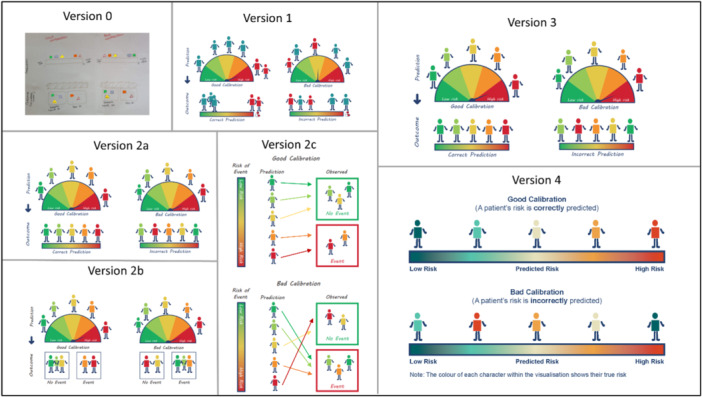
Progression of visualising the term ‘calibration’.

To provide a starting point for the graphic designer, the working group met in person and collaboratively sketched some ideas of how to visualise the statistical methodology terms (see some examples [versions 0] in Figures 4, 5, 6, 8, and 9). Some terms were easier to instinctively conceptualise visualisations (e.g., censoring, regression, and deviance) than other terms that were more complex (e.g., calibration and simulation study). For ‘simulation study’, the working group struggled to create a coherent sketch, so the graphic designer created a first version based solely on the written definition (see v1 in Figure 3). Once the graphic designer had produced a first version of all 10 terms (see some examples [versions 1] in Figures 3, 4, 5, 6, 8, and 9), the working group provided some initial feedback. This ranged from changing wording and layout to complete redesigns. For example, the components and layout for ‘prognostic model’ were completely changed (v2a vs. v1 in Figure 4), whereas for ‘discrimination’, the only amendments were changing the colouring of some of the characters and removing the dial needle and pills symbol (v2 vs. v1 in Figure 5).

The working group still did not feel that the visualisation for ‘simulation study’ was a helpful visual aid in its current form (v2a of Figure 3). Before any further PPIE meetings, the working group considered the visualisation and written definition together. Originally, the written definition was ‘A simulation study uses a computer program to learn about different situations to understand what might happen, instead of doing a real‐world experiment. It's like getting a computer to see which route to work is the best by “simulating” different traffic conditions, instead of trialling them all yourself’. This was subsequently revised to explain that simulation studies can be used to select an appropriate statistical tool to analyse data and included the analogy of selecting an appropriate tool to dig a hole. This analogy was previously included in the PPI‐SMART animation which explains statistical methodology [7]. The ‘digging a hole’ example used in the revised definition was then used as a basis for developing a new visualisation sketch to support the definition (v2b of Figure 3).

### 3.4 Stage 4: Cycles of PPIE Feedback

#### 3.4.1 February Meeting

In February 2025, we reviewed the visualisations and held an online PPIE meeting with PPIE Group 1 to gather feedback on four of the most challenging visualisations, or those in which we had multiple versions. These included: Simulation Study (v2b of Figure 3), Prognostic model (v2 of Figure 4), Discrimination (v2 of Figure 5) and Calibration (v2 of Figure 6).

When the public contributors were shown the ‘digging a hole’ visualisation for ‘simulation study’ (v2b of Figure 3), they expressed that they liked the use of this concept since they remembered it from the animation and it provided consistency, but that the drawing of the hole was not clear and should be updated to match the imagery used in the animation. To emphasise that a simulation study uses computer inputs, it was suggested that the different tool options ‘pass through’ the computer, rather than being seen through magnifying glasses. Furthermore, group members suggested visualising the chosen model/method coming out of the computer.

We presented two visualisations to the group to explain a prognostic model. Whilst both had limitations, the group provided valuable feedback suggesting making it clearer within the visualisation that a prognostic model analyses data. They advised against using symbols with potential positive or negative connotations (e.g., an apple for ‘good nutrition’), recommending instead the use of neutral imagery such as test results or thermometers. The group responded positively to the use of grey figures in v2a (Figure 4), which changed colour to indicate the prognostic model had calculated each individual's risk. The group found the conveyor belt concept in v2b easier to follow overall. Suggestions for improving v2b to aid understanding of the visualisation included incorporating the changing colours as done in v2a and including supporting text such as ‘What is my risk?’ rather than question marks and speech bubbles above peoples' heads. It was suggested though that a conveyer belt felt ‘mechanical’ and ‘forced’, and so walking along a path was chosen instead (compare v3 with v2b in Figure 4).

There was discussion regarding differentiating between model accuracy (calibration) and how well the model can separate groups (discrimination). This resulted in adjusting the visualisation for discrimination to incorporate risk groups (v3 vs. v2 in Figure 5). The term ‘good discrimination’ used within the visualisation for discrimination was found to be confusing. The group highlighted that the word ‘discrimination’ carries a different and often negative meaning in everyday language compared to its technical use in statistics. Consequently, this text was temporarily removed, with the intention of refining and reintroducing it at a later date, using language such as ‘separating groups’ instead. The glossary definition for ‘discrimination’ was also updated to emphasise that this term has a different meaning in statistics than it does in everyday language. The group responded positively to the visualisation options for calibration, commenting that using traffic light colours to indicate levels of risk effectively conveyed those at high and low risk. The third option (v2c of Figure 6) was the least preferred and considered to be too difficult to understand due to having to read downwards and the many arrows present. Regarding the other two options, some liked the box options in v2b, whilst others felt v2a was easier to understand. Some members suggested that some of the words chosen (e.g., prediction) were of too high a reading age; however, the working group found it hard to find suitable alternative words. The working group decided to take v2a forward to the next PPIE meeting.

#### 3.4.2 March Meeting

In March 2025, we met online with PPIE Group 2 to review the latest versions of all 10 visualisations (examples are versions 2 or 3 [if present] of Figures 3, 4, 5, 6, 8, and 9). Positive feedback included how some visualisations helped explain the harder parts of the respective definition. The visualisations that were clear and simply explained were well received. In addition to specific feedback for certain visualisations, the group gave general feedback about the accessibility of the visualisations.

**Figure 7:**
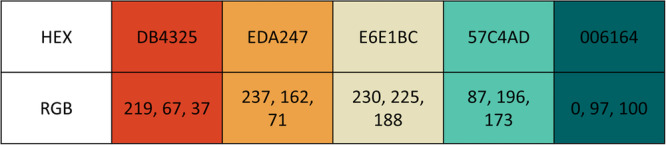
Suggested colour palette that is colour‐blind friendly (HEX and RGB code)—credit to Andy Kirk.

Whilst the traffic light colour scheme was favoured by PPIE Group 1, it was highlighted that it would not be accessible for anyone with red‐green colour blindness. As such, an alternative colour palette was found that would be suitable for people with red‐green colour blindness, but still indicate levels of risk. The colour palette in Figure 7 was chosen, an alternative palette for red‐green colour blindness suggested by Andy Kirk, which keeps the connotation of a red hue being perceived as ‘bad’ [12]. In addition, the group highlighted the need to consider the colour of text if using a coloured background and the choice of font so that the text can be read by those with visual impairments or dyslexia.

Consistency within and across definitions was suggested. For the simulation study, changing a decorator's trowel to a gardening trowel reduced confusion as now all items would be related to gardening or digging (v4 vs. v3 in Figure 3). For the prognostic model, having the same number of people going ‘in’ and ‘out’ of the ‘model’ was preferred to reduce confusion (v4 vs. v3 in Figure 4).

**Figure 8:**
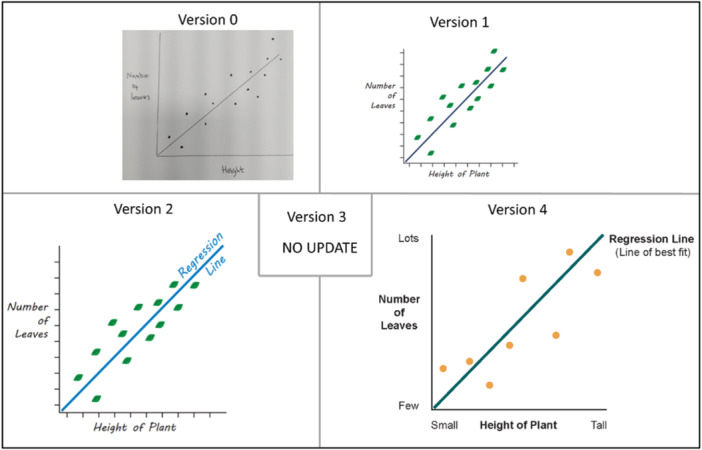
Progression of visualising the term ‘regression’.

**Figure 9:**
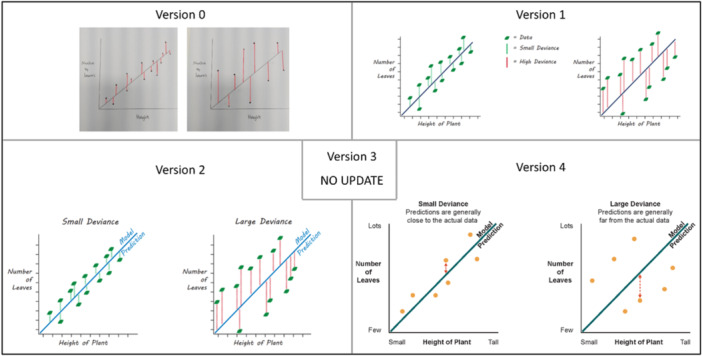
Progression of visualising the term ‘deviance’.

Familiarity with words, symbols and graphs was highlighted amongst multiple visualisations. For the prognostic model, the phrase ‘Data Analysis’ may not be understood by everyone (v3 in Figure 4). For regression and deviance, both visualisations feature a graph (v2 in Figures 8 and 9). As researchers, we thought the simplified drawing of a graph would be familiar to most people; however, members of PPIE Group 2 highlighted that this is not the case and that there are many people who would not have the background to know how to interpret a graph. As such, we were encouraged to amend elements to help understand the graph layout, such as removing the ticks, but adding the words ‘few’ and ‘lots’ along the axes. We were also encouraged to keep things simple and avoid overinterpretation by using simple dots on the graph, rather than the leaves that were originally added to link with the example around plants. For deviance, it was felt that including lines between every data point and the regression line was confusing and overwhelming for the viewer. To simplify, we instead included one arrow and added some text to clarify that deviance relates to how close the model predictions are to the actual data (compare v4 against v2 in Figures 8 and 9). For causal inference, the members of the group found that they were over‐interpreting the symbols chosen for ‘exercise’, focusing on the specific activity represented rather than exercise in general. In the spirit of simplifying elements of the visualisations, the working group also decided to remove any remaining dials and scales in the visualisations for discrimination and calibration.

### 3.5 Stage 5: Release

**Figure 10:**
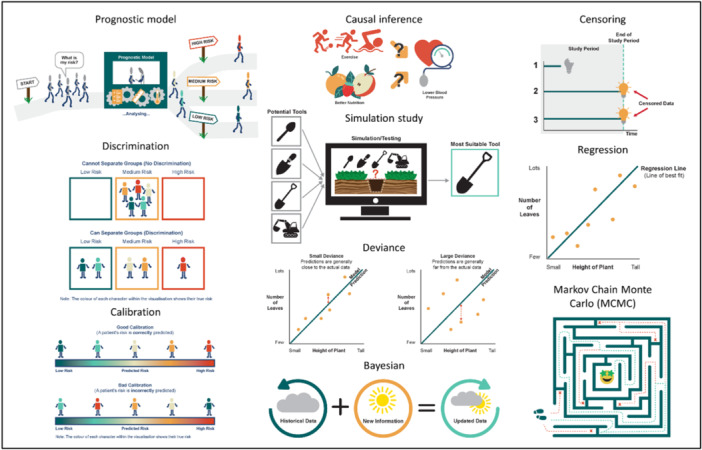
Final visualisations for the chosen 10 terms.

The final visualisations can be viewed in Figure 10 and have been made freely available on the NIHR Leicester Biomedical Research Centre (BRC) website (https://leicesterbrc.nihr.ac.uk/ppismart/ppismart-definitions/) for researchers to use.

## 4 Discussion

To support PPIE activities in future statistical methodology research projects, we developed public‐friendly visualisations for 10 definitions from our plain‐language glossary. The visualisations are freely available online for researchers to use, to improve and aid PPIE within research involving statistical methodology. The activities described in this manuscript can be seen as the beginning of creating useful resources for PPIE, especially for definitions that are hard to understand with only words. Additionally, these visualisations will be particularly useful for PPIE contributors who do not typically take part in research related to methodology, as they provide accessible explanations of complex statistical concepts.

Naturally, as with the plain‐language glossary, we focused on terms that are relevant to the research fields that members of the PPI‐SMART team often work within, as well as terms suggested by the PPIE groups. As such, this has resulted in the first set of visualisations focusing on areas such as prognostic modelling, statistical modelling in general, and survival analysis. There exist other areas of statistical methodology research, for which these visualisations would not necessarily benefit related PPIE activities.

We worked with two PPIE groups with differing backgrounds and experiences with statistical methodology research. Working with two PPIE groups that had varying levels of experience with statistical methodology research was beneficial to the project, enabling us to get a wider view and opinions of the visualisations and making them public‐friendly. However, we could have broadened our PPIE activities further. Specifically, all the members of our PPIE groups were already aware of the idea of PPIE and had previously been involved in the development of our plain language glossary—it may have been beneficial to gain feedback on our visualisations from people who are totally new to PPIE and/or the broader project. Also, whilst the PPIE groups had a mixture of protected characteristics, we were not able to achieve fully inclusive recruitment to capture the full range of PPI perspectives.

There were some instances where a balance had to be struck regarding the language used in the visualisation. For some visualisations, PPIE members suggested changing/removing certain words due to being seen as too high‐level; however, the statistical researchers on the team felt that some words could not be changed due to their inherent definition in the related field. For example, one of our terms was ‘discrimination’. As mentioned in the results, the PPIE members raised the fact that discrimination has another meaning in general society and questioned whether it was ‘needed’. However, whilst we made sure to be careful with how the word was used, it was ultimately kept in the visualisation as ‘discrimination’ is the technical term for assessing a prognostic model and has no recognised alternative name.

Overall, the PPIE work conducted in this project was essential and highly impactful—allowing us to ensure that our visualisations were understandable to members of the public. As researchers, it is very easy to become unaware of what information is specialist or known to all—carrying out PPIE activities ensured that our work is accessible to as many people as possible. To ensure that this work continues to be impactful, the existence of the visualisations will be disseminated across multiple formats. As well as being freely available on our website—along with our other resources—we will promote these visualisations through various communication channels that have previously proven effective across multiple research groups. Specifically, this will include social media, uploading to resource platforms (e.g., Learning for Involvement), webinars, and peer networks such as the PPI for Statisticians Community of Practice group.

In future, it would be beneficial to gain feedback on the use of the visualisations—whether they helped engagement or whether certain elements need improving to aid understanding. We would also welcome the opportunity to develop more visualisations for other definitions in our glossary that may benefit from a visual aid, as well as inviting others to consider developing visualisations for their specific field within statistical methodology research.

Whilst other glossaries exist [9], to our knowledge, this is the first project to develop and make available visualisations to aid PPIE activities regarding complex terminology. Other tools have been developed to support PPIE activities, such as an involvement matrix to allow researchers and PPIE contributors to discuss and explain the PPIE roles within a project [13], as well as various frameworks or ‘toolkits’ to help guide researchers through the various processes related to PPIE [11, 14, 15]. By enabling clearer understanding through these developed visualisations, PPIE contributors are better equipped to question, influence and contribute to decisions being made in statistical methodology research. This enhanced involvement supports the development of statistical methods that are more likely to facilitate analyses that lead to actionable and equitable health insights. In this way, improving accessibility within research involving statistical methodology creates upstream benefits that can, over time, contribute to more robust evidence bases, more relevant analyses, and ultimately improved health outcomes and policies.

## 5 Conclusion

Whilst glossary definitions of complex terms are a useful resource for PPIE activities, some terms lend themselves to being explained more easily through visual methods. We developed 10 public‐friendly visualisations to improve accessibility and support PPIE in the development and application of statistical methodology. By helping to embed PPIE more effectively within such projects, this work aims to contribute to improved health outcomes and policies.

## Author Contributions

**Clareece R. Nevill:** conceptualisation (equal), investigation (supporting), visualisation (equal), writing – original draft (lead), writing – review and editing (equal). **Hannah Cooper:** investigation (supporting), visualisation (equal), writing – original draft (supporting), writing – review and editing (equal). **Barbara Czyznikowska:** investigation (lead), writing – review and editing (equal). **Louise Fairgrieve:** resources (lead), visualisation (equal), writing – review and editing (equal). **Justin Greenwood:** visualisation (equal), writing – review and editing (equal). **Gurpreet Grewal‐Santini:** investigation (supporting), writing – review and editing (equal). **Janion Nevill:** visualisation (equal), writing – review and editing (equal). **Lucy Teece:** conceptualisation (equal), visualisation (equal), writing – review and editing (equal). **Molly Wells:** conceptualisation (equal), visualisation (equal), writing – review and editing (equal). **Suzanne C. Freeman:** conceptualisation (equal), writing – review and editing (equal). **Sarah Booth:** conceptualisation (equal), investigation (lead), visualisation (equal), writing – original draft (supporting), writing – review and editing (equal).

## Disclosure

The views expressed are those of the author(s) and not necessarily those of the NIHR or the Department of Health and Social Care.

## Ethics Statement

The authors have nothing to report.

## Consent

The authors have nothing to report.

## Conflicts of Interest

L.T. is an employee of AstraZeneca and has stock ownership and/or stock options or interests in the company. S.B. was employed by Aviva for work unrelated to this research during the peer review of this manuscript. All other authors declare that they have no competing interests.

## Clinical Trial Registration

Not applicable.

## Permission to Reproduce Material From Other Sources

Not applicable.

## Supporting information


**Table S1:** GRIPP2 Short Form.

## Data Availability

Our visualisations are available online at https://leicesterbrc.nihr.ac.uk/ppismart/ppismart-definitions/. Data sharing is not applicable to this article as no datasets were generated or analysed during the current study.
