# Supplementary material for: Developing Public‐Friendly Visualisations to Improve PPIE Glossaries for Statistical Methodology Research
Source: Health Expect. 2026 May 14;29(3):e70690. doi: 10.1111/hex.70690 (PMC13176640; doi:10.1111/hex.70690)
Supplement: Supplementary file 1 — Table S1: GRIPP2 Short Form. [file HEX-29-e70690-s001.docx]

**Table 1: GRIPP2 Short Form**

| **Section and topic** | **Item** | **Reported on page No** |
| --- | --- | --- |
| 1. Aim | Report the aim of PPI in the study | 2 |
| 1. Methods | Provide a clear description of the methods used for PPI in the study | 3-4 |
| 1. Study results | Outcomes – Report the results of PPI in the study, including both positive and negative outcomes | 4-10 |
| 1. Discussion and conclusions | Outcomes – Comment on the extent to which PPI influenced the study overall. Describe positive and negative effects. | 12 |
| 1. Reflections/critical perspective | Comment critically on the study, reflecting on the things that went well and those that did not, so others can learn from this experience | 12 |
